# The microbiota-metabolite-immune axis in the olfactory cleft microenvironment: mechanisms and therapeutic implications for dysbiosis-driven olfactory dysfunction in chronic rhinosinusitis

**DOI:** 10.3389/fimmu.2026.1841979

**Published:** 2026-07-07

**Authors:** Jin-Xiang Zhu, Guan-Jiang Huang

**Affiliations:** 1 Department of Otorhinolaryngology Head and Neck Surgery, Zhongshan Hospital of Traditional Chinese Medicine, Affiliated to Guangzhou University of Chinese Medicine, Zhongshan, Guangdong, China; 2 The Tenth Clinical Medical College of Guangzhou University of Chinese Medicine, Zhongshan, Guangdong, China

**Keywords:** chronic rhinosinusitis, metabolomics, microbiota-metabolite-immune axis, nasal microbiome, olfactory cleft, olfactory dysfunction, *Staphylococcus aureus*, type 2 inflammation

## Abstract

Chronic rhinosinusitis is the leading cause of olfactory dysfunction in adults. Although mechanical obstruction and type 2 inflammation remain important explanations for smell loss in chronic rhinosinusitis, emerging multi-omics studies suggest that disruption of the olfactory cleft microenvironment may also contribute to olfactory dysfunction. In this review, we propose the microbiota-metabolite-immune (MMI) axis as an integrative framework linking microbial dysbiosis, metabolite perturbation, and local immune remodeling in CRS-associated olfactory dysfunction. We systematically examine four interconnected domains. First, several studies have reported dysbiosis within the olfactory niche, including enrichment of *Acinetobacter johnsonii* in one CRS-OD cohort together with depletion of putative commensals. Second, altered metabolite profiles in CRS-OD have been associated with disturbed purine metabolism, uric acid accumulation, and reduced levels of the potentially protective metabolite indole-3-acetic acid. These changes may contribute to innate inflammatory signaling, including Toll-like receptor 4/nuclear factor kappa-light-chain-enhancer of activated B cells (TLR4/NF-κB)-related pathways. Third, *Staphylococcus aureus* superantigens may promote T helper 2 (Th2) polarization, alter regulatory T-cell function, disrupt tight junction integrity, and impair olfactory neurogenesis, thereby sustaining bidirectional immune-microbial crosstalk. Fourth, emerging microbiota-targeted therapeutics, including xylitol irrigation, probiotics, and Interleukin-4 receptor alpha (IL-4Rα) blockade, offer novel intervention strategies. Throughout this review, we distinguish olfactory cleft-specific evidence from broader sinonasal data and acknowledge the current predominance of association studies over causal validation. Taken together, the MMI axis provides a useful framework for understanding CRS-associated OD and for identifying testable therapeutic hypotheses.

## 1 Introduction

The olfactory cleft (OC) is a narrow anatomical corridor bordered superiorly by the cribriform plate, medially by the nasal septum, and laterally by the middle turbinate and superior turbinate. Its mucosal lining comprises a pseudostratified olfactory neuroepithelium (OE) uniquely distinct from the adjacent respiratory mucosa, housing olfactory sensory neurons (OSNs), sustentacular cells, microvillar cells, and horizontal and globose basal stem cells embedded within a Bowman’s glands-derived mucus layer rich in odorant-binding proteins (1–3). Chronic rhinosinusitis (CRS) is a highly prevalent inflammatory sinonasal disease affecting approximately 12% of the global adult population and constitutes the primary cause of olfactory dysfunction (OD) in young and middle-aged adults (1, 4, 5). Quantifiable OD affects 60% to 83% of patients with CRS and ranks among the most distressing and treatment-refractory symptoms of the disease (6, 7).

Historically, CRS-associated OD has been attributed to two principal mechanisms: conductive loss arising from polyp-mediated obstruction of olfactory airflow, and sensorineural loss arising from inflammatory cytokine-mediated injury to the OE (1, 8). The neuroepithelial basis of sensorineural loss was established in early histopathological work demonstrating that olfactory mucosa from patients with chronic rhinosinusitis harbors inflammatory infiltrates of lymphocytes, macrophages, and eosinophils, with severity correlating with objective smell scores. However, a substantial proportion of patients maintain olfactory loss despite preserved OC patency or following surgical polyp removal, and objective smell scores often fail to fully recover even after endoscopic sinus surgery (2, 4, 9–12). This persistent, surgery-refractory olfactory impairment suggests that mechanisms beyond gross anatomical obstruction must be operative.

The emergence of culture-independent metagenomics, untargeted metabolomics, and high-dimensional cytokine profiling has revealed a previously underappreciated layer of CRS pathobiology: the microbial and metabolic milieu of the olfactory niche (6, 13–15). Recent multi-omics studies have reported differences in nasal microbiome composition, metabolite profiles, and local inflammatory mediators between CRS patients with and without OD. These convergent findings support an integrated microbiota-metabolite-immune (MMI) axis framework in which microbial dysbiosis drives metabolite perturbations that amplify immune responses capable of directly injuring olfactory neuroepithelium, impairing OSN neurogenesis, and perpetuating a self-reinforcing pathological cycle.

To our knowledge, this is the first systematic review to integrate multi-omics, mechanistic, and clinical trial data into a unified pathobiological framework for the MMI axis in the OC microenvironment. This framework is intended to complement, rather than replace, the established eosinophilic and type 2 inflammation paradigm by highlighting the possibility that microbial and metabolic disturbances may represent an additional layer of disease biology in CRS-OD. Throughout, findings directly obtained from olfactory cleft sampling are distinguished from those derived from the middle meatus or broader sinonasal compartment, given the ecological distinctiveness of these niches. Throughout, we explicitly acknowledge that the available evidence is predominantly associative rather than mechanistic, and we identify corresponding research gaps as priority areas for future investigation.

**Figure 1:**
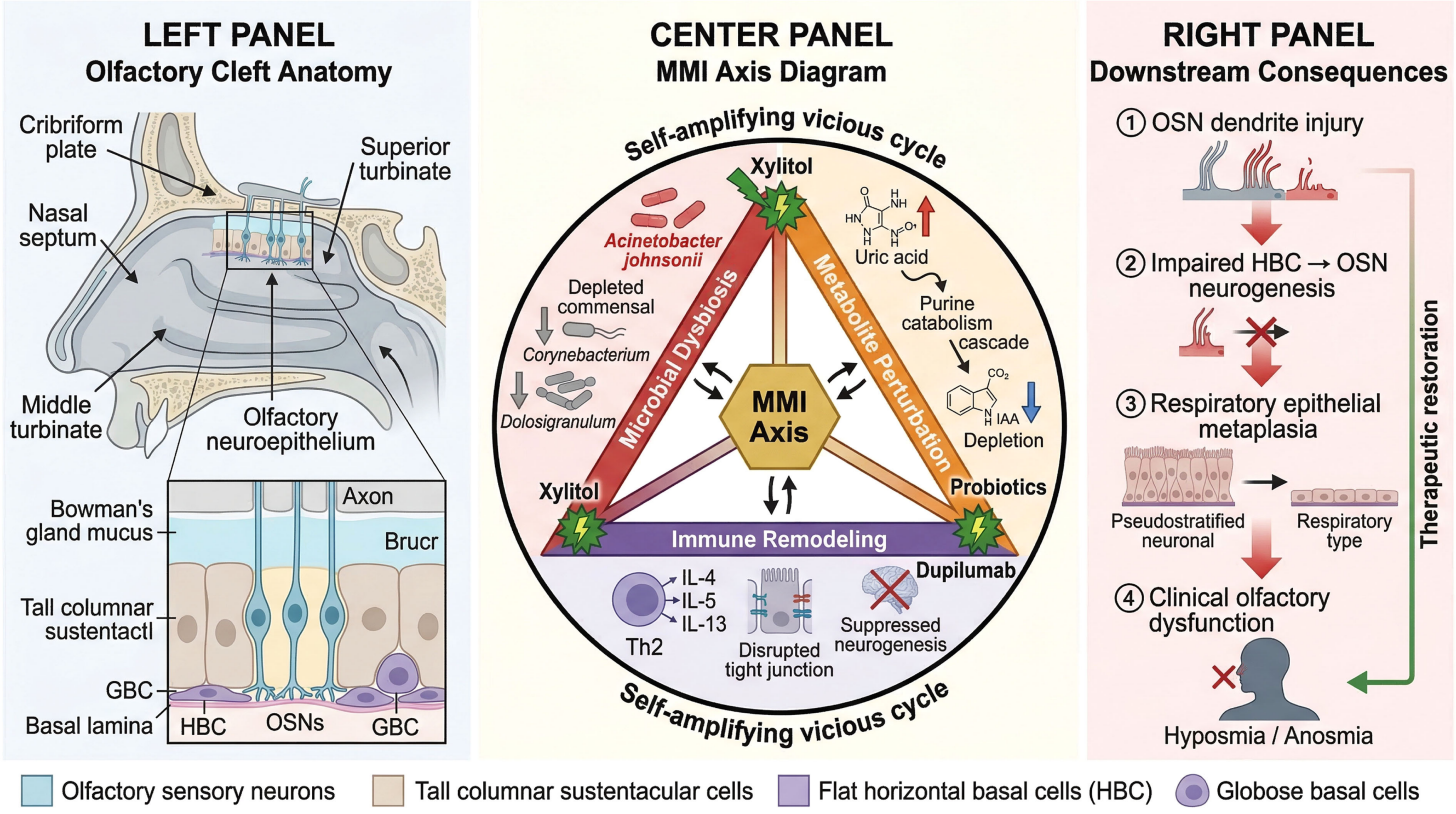
Schematic overview of the olfactory cleft microenvironment and the microbiota-metabolite-immune (MMI) axis. This schematic illustrates the anatomical basis of the olfactory cleft (OC) niche, the core architecture of the microbiota-metabolite-immune (MMI) axis, and its downstream pathological cascade driving olfactory dysfunction. Left panel: Anatomical diagram of the OC niche, bordered superiorly by the cribriform plate, medially by the nasal septum, and laterally by the superior and middle turbinates. The olfactory neuroepithelium (OE), containing olfactory sensory neurons (OSNs), sustentacular cells, and horizontal basal cells, is highlighted. Central panel: The three interconnected arms of the MMI axis: microbial dysbiosis (Arm 1), metabolite perturbation (including purine metabolite accumulation and indole-3-acetic acid (IAA) depletion, Arm 2), and immune remodeling (including T helper 2 (Th2) polarization and tight junction disruption, Arm 3). Right panel: Downstream consequences of MMI axis dysregulation on olfactory sensory neurogenesis and clinical olfactory dysfunction. Therapeutic intervention points targeting the MMI axis are highlighted in green.

This review synthesizes evidence from multi-omics clinical studies, mechanistic animal models, and clinical trials to characterize the MMI axis in CRS-OD and identify therapeutic targets for restoring olfactory homeostasis. The anatomical basis of the olfactory cleft niche, the core architecture of the MMI axis, and its downstream pathological cascade leading to olfactory dysfunction are comprehensively illustrated in **Figure 1**.

## 2 The olfactory cleft as a distinct microbial niche

### 2.1 Baseline microbial ecology of the healthy olfactory area

The nasal cavity harbors a complex, region-specific microbial ecosystem shaped by host immunology, mucosal physiology, and environmental exposure. Under healthy conditions, the dominant commensal genera include *Corynebacterium*, *Dolosigranulum*, coagulase-negative *Staphylococcus*, and *Propionibacterium*, which collectively maintain mucosal homeostasis through competitive exclusion, bacteriocin production, and immune education (16–19) The olfactory area is the most superior and anatomically confined region of the nasal vault. It harbors a complex microbiome community that mirrors and potentially shapes olfactory function. As demonstrated by *Koskinen* et al., microbial community composition differs significantly among individuals with normosmia, hyposmia, and anosmia, even in the absence of overt sinonasal disease (13).

During healthy homeostasis, mitotically active globose basal cells (GBCs) continuously replenish mature OSNs throughout adult life, while quiescent horizontal basal cells (HBCs) serve as the emergency reserve for OE regeneration following severe injury. Single-cell RNA sequencing of the human OC mucosa by *Durante* et al. identified active neurogenic progenitor pools capable of sustaining OSN production for decades, establishing the OC as a lifelong neurogenic niche (20–23). This regenerative biology is likely vulnerable to disruption by chronic inflammatory signals arising from the local mucosal environment.

### 2.2 Microbiome alterations associated with CRS-OD

Multiple clinical microbiome studies have reported differences between CRS patients with olfactory dysfunction, although direct comparison across studies is complicated by variation in sampling site, sequencing platform, and patient phenotype. We systematically summarized the key clinical and microbiological studies investigating the association between sinonasal microbial dysbiosis and CRS-related OD in **Table 1**, with explicit distinction between OC-specific and general sinonasal sampling findings. In a prospective shotgun metagenomic study of 63 patients with CRS, *Han* et al. reported lower alpha diversity in patients with olfactory dysfunction than in those without olfactory dysfunction (6). Linear Discriminant Analysis Effect Size (LEfSe) analysis revealed significant enrichment of *Acinetobacter johnsonii* (Linear Discriminant Analysis score > 3) in the OD group, whereas *Mycoplasma arginini*, *Aeromonas dhakensis*, and *Salmonella enterica* were significantly depleted (6). *Acinetobacter* species are recognized opportunistic pathogens with robust biofilm-forming capacity and intrinsic antimicrobial resistance. Because this observation was derived from a single cohort, the biological significance of *A. johnsonii* enrichment in the olfactory cleft remains to be established experimentally. Several 16S rRNA-based studies have associated lower abundance of *Corynebacterium* and *Dolosigranulum* with greater sinonasal inflammatory burden, including CRS subgroups with olfactory impairment. Such organisms may limit pathobiont overgrowth through competition for mucosal attachment sites, production of bacteriocins, and modulation of local innate immune tone. Whether their depletion in CRS is a primary driver of disease or a secondary consequence of the inflammatory milieu remains incompletely resolved, and prospective microbiome intervention studies are needed to address this question (16, 24–26).

**Table 1:** Key clinical and multi-omics-based studies of the nasal and olfactory cleft microbiome in olfactory dysfunction with CRS.

First author (year)	Journal	Study design	n (OD/control)	Sampling site	Method	Key microbial finding	Olfactory assessment	Main result
Han X et al. (2023) (6)	Front Immunol	Prospective cohort	63 CRS (OD vs NOD)	Nasal mucus	Shotgun metagenomics	↑ Acinetobacter johnsonii; ↓ Mycoplasma arginini, Aeromonas dhakensis	Sniffin’ Sticks TDI	OD group showed decreased diversity; dysbiosis correlated with purine metabolite elevation and cytokine increases
Kidoguchi M et al. (2023) (24)	J Allergy Clin Immunol	Cross-sectional	67 eosinophilic CRS	Middle meatus	16S rRNA	↓ Lactobacillus; ↑ gram-negative pathobionts in eosinophilic CRS	Not reported	Eosinophilic CRS endotype associated with distinct microbiome profile distinct from non-eosinophilic CRS
Lim SJ et al. (2023) (104)	Microorganisms	Cross-sectional	Pediatric CRS cohort	Nasal swab	16S rRNA	Decreased diversity with increasing age and severity	UPSIT	Microbial diversity inversely correlates with CRS severity markers

This table systematically summarizes landmark clinical and multi-omics-based studies investigating the association between sinonasal microbial dysbiosis and olfactory dysfunction (OD) with chronic rhinosinusitis (CRS). Findings from olfactory cleft-specific sampling are explicitly distinguished from those derived from the middle meatus or general sinonasal compartment in the “Sampling Site” column. NOD, non-olfactory dysfunction; TDI, Threshold, Discrimination, Identification; UPSIT, University of Pennsylvania Smell Identification Test.

In an olfactory area-specific study using 16S rRNA sequencing, metatranscriptomics, and propidium monoazide treatment, *Kumpitsch* et al. reported higher alpha diversity in dysosmic participants and inverse associations between olfactory performance and oral-associated taxa (14). Alpha diversity was paradoxically elevated in dysosmic subjects, driven by atypical taxa including *Rickettsia*, *Spiroplasma*, and *Brachybacterium*, while olfactory performance was negatively associated with microbial signatures from the oral cavity and periodontitis, including *Fusobacterium*, *Porphyromonas*, and *Selenomonas*. Propidium monoazide treatment further revealed a significantly higher burden of dead microbial biomaterial in dysosmic subjects, indicating impaired mucosal clearance mechanisms (14). In the eosinophilic CRS endotype, which predominates in East Asian populations and is most strongly associated with OD, *Kidoguchi* et al. demonstrated distinct middle meatus microbiome profiles with reductions in *Lactobacillus* and expansions of gram-negative pathobionts (24). Because this study analyzed middle meatus samples rather than olfactory cleft samples, it is better interpreted as evidence for broader sinonasal microbiome remodeling in eosinophilic CRS rather than direct proof of olfactory cleft-specific dysbiosis.

A critical methodological consideration is that the majority of microbiome studies cited in this section were conducted in the middle meatus rather than the olfactory cleft proper, with the notable exceptions of the olfactory area-specific investigations by *Kumpitsch* et al. and *Koskinen K* (13, 14). These data are informative but should not be assumed to reproduce the ecology of the OC exactly. In addition, the available evidence is predominantly observational; direct causal links between specific taxa and olfactory epithelial injury remain to be demonstrated.

### 2.3 Nasal dysbiosis and the olfactory-neurological axis

Emerging evidence from the Translational Psychiatry study by *Song H* et al. links nasal microbiome perturbations not only to olfactory dysfunction but also to cognitive decline in older adults (27). Deep nasal sinus cavity microbiota dysbiosis has been documented in patients with Parkinson’s disease, with the olfactory bulb serving as a direct anatomical conduit between nasal microbial communities and the central nervous system (28–30). While a comprehensive treatment of the nasal-brain axis is beyond the scope of this review, these findings highlight that olfactory niche dysbiosis may carry systemic neurological consequences extending well beyond local sinonasal pathology (27, 28, 31).

### 2.4 Polymicrobial interactions and the protective role of commensals

Although *Staphylococcus aureus* is highly relevant to the MMI axis, chronic rhinosinusitis is more appropriately viewed as a polymicrobial disorder than as a single-organism disease. Pathobionts frequently engage in complex interspecies interactions within the sinonasal cavity. For example, *Staphylococcus aureus* often co-exists with *Pseudomonas aeruginosa* or *Haemophilus influenzae* in mixed-species biofilms. Mixed-species biofilms involving *Staphylococcus aureus*, *Pseudomonas aeruginosa*, and *Haemophilus influenzae* may show greater persistence and immune evasion than monospecies communities. Conversely, commensal organisms such as *Corynebacterium* and *Dolosigranulum* provide crucial protective functions.Such organisms may limit pathobiont overgrowth through competition for mucosal attachment sites, production of bacteriocins, and modulation of local innate immune tone. Genera such as *Corynebacterium* and *Dolosigranulum* have been associated with mucosal homeostasis and competitive exclusion of pathobionts, although their protective roles in the olfactory cleft specifically still require direct experimental confirmation (32).

## 3 Metabolite-mediated immune remodeling in the olfactory niche

### 3.1 Multi-omics characterization of the CRS-OD metabolome

The metabolome of OC mucus reflects the combined biochemical output of host cells and resident microbiota and may provide a useful readout of the local microenvironment (15, 33). Combined Liquid Chromatography-Mass Spectrometry (LC-MS) and Gas Chromatography-Mass Spectrometry (GC-MS) profiling by *Han* et al. identified 21 differential nasal metabolites in OD versus NOD patients, with significant elevations in uric acid, 4-aminophenol, 2’-deoxyguanosine, and inosine, and notable reductions in ascorbic acid and IAA. The detailed information of these key differential metabolites, including their metabolic classification, change trend, statistical significance, and functional annotations, is comprehensively listed in **Table 2**. Kyoto Encyclopedia of Genes and Genomes (KEGG) pathway enrichment analysis demonstrated that differential metabolites converged on purine metabolism as the most significantly perturbed subpathway in OD patients (*P* < 0.001), followed by arginine and proline metabolism, sphingolipid signaling, cyclic Adenosine Monophosphate (cAMP) signaling, and neuroactive ligand-receptor interaction (6).

**Table 2:** Key differential nasal mucus metabolites between CRS patients with and without olfactory dysfunction.

Metabolite	Metabolic pathway	Direction in CRS-OD	Detection method	Statistical significance	Proposed biological function
Uric acid	Purine metabolism	Elevated	GC-MS	P < 0.05	Damage-Associated Molecular Pattern (DAMP) activating TLR4/NF-κB; the NOD-like receptor family pyrin domain containing 3 (NLRP3) inflammasome agonist; promotes M1 macrophage polarization
Inosine	Purine metabolism	Elevated	GC-MS	P < 0.05	Purine nucleoside; precursor in uric acid production pathway
2’-Deoxyguanosine	Purine metabolism	Elevated	GC-MS	P < 0.05	Purine nucleoside; marker of DNA oxidative stress
4-Aminophenol	Amino acid metabolism	Elevated	GC-MS	P < 0.05	Pro-inflammatory amine derivative
Indole-3-acetic acid (IAA)	Tryptophan metabolism	Depleted	LC-MS	P < 0.05	AhR ligand; suppresses macrophage TNF-α, IL-1β, MCP-1; anti-inflammatory and neuroprotective
Ascorbic acid	Antioxidant metabolism	Depleted	GC-MS	P < 0.05	Antioxidant; supports collagen synthesis; reduces oxidative OSN injury
4-Aminobutyric acid	Neuroactive ligand pathway	Altered	GC-MS	P < 0.05	Inhibitory neurotransmitter; modulates olfactory signal transduction
O-Phosphoethanolamine	Phospholipid metabolism	Altered	GC-MS	P < 0.05	Phospholipid precursor; membrane integrity
Agmatine	Arginine metabolism	Altered	GC-MS	P < 0.05	Polyamine precursor; neuromodulator

This table lists core differential nasal metabolites identified via combined Liquid Chromatography-Mass Spectrometry (LC-MS) and Gas Chromatography-Mass Spectrometry (GC-MS) by Han et al. (2023), in CRS patients with olfactory dysfunction (CRS-OD) compared to CRS patients without olfactory dysfunction (CRS-NOD). The metabolic classification, change trend, statistical significance, and proposed biological function of each metabolite are detailed. IAA, indole-3-acetic acid; DAMP, damage-associated molecular pattern; TLR4, Toll-like receptor 4; NF-κB, nuclear factor kappa-light-chain-enhancer of activated B cells; NLRP3, NOD-like receptor family pyrin domain containing 3; OSN, olfactory sensory neuron; AhR, aryl hydrocarbon receptor; TNF-α, tumor necrosis factor-alpha; IL-1β, interleukin-1 beta; MCP-1, monocyte chemoattractant protein-1.

Importantly, these metabolomic findings originate from nasal mucus samples and represent clinical association data generated within a single prospective cohort. Direct causal evidence that the identified metabolite perturbations are sufficient to induce olfactory epithelial injury within the olfactory cleft microenvironment awaits confirmation in olfactory niche-targeted animal models.

### 3.2 The uric acid-TLR4/NF-κB pathway and innate immune activation

Purines fulfill fundamental roles in cellular energy homeostasis, olfactory signal transduction via purinergic receptors, neuromodulation, and OE maintenance (34). In the CRS cohort reported by *Han* et al., uric acid was elevated in nasal mucus from patients with olfactory dysfunction. Outside the olfactory cleft, excess uric acid has been shown to function as a danger-associated signal in inflammatory settings. At supraphysiological concentrations, uric acid engages TLR2 and TLR4 on airway epithelial cells and resident macrophages via the URAT1 transporter, activating the NF-κB pathway and driving the production of Tumor Necrosis Factor-alpha (TNF-α), Interleukin-1 beta (IL-1β), and Interleukin-6 (IL-6), while promoting M1 macrophage polarization (35–38). Concurrently, uric acid crystal microdeposits activate the NLRP3 inflammasome, amplifying local IL-1beta output in a caspase-1-dependent manner (39). Independent experimental work has also shown that pro-inflammatory cytokines can impair olfactory function in the absence of purely conductive obstruction. Lane and colleagues reported that chronic tumor necrosis factor-alpha (TNF-α) signaling suppresses odor-evoked responses, impairs regeneration, and promotes neuronal injury, whereas chronic interferon-gamma (IFN-γ) expression reduces odor responsiveness without overt neuroepithelial destruction. Together, these cytokine-mediated mechanisms rapidly impair olfactory signal transduction and deplete the neuroepithelial reserve, representing pathological pathways that converge with the MMI axis-driven metabolite and dysbiosis arms to compound olfactory injury (40, 41).

This innate immune cascade creates a self-reinforcing inflammatory circuit that is capable of injuring sustentacular cells and OSN cilia independently of adaptive immune mechanisms. The convergence of uric acid-driven innate activation with documented accumulation of eosinophil-derived neurotoxin (EDN) in OC mucus argues that dysbiosis-induced metabolite perturbation amplifies established type 2 pathways in the olfactory niche. In the study by *Wu* et al., mucus EDN levels independently predict OD with an Area Under the Curve (AUC) = 0.873 compared to 0.764 for blood eosinophil counts (*P* = 0.024) (42). In a related line of evidence, *Xu* et al. demonstrated that abnormal purine metabolism in nasal epithelial cells regulates the T helper 17/Regulatory T cell (Th17/Treg) balance, suggesting that purine catabolism dysregulation may carry multi-axis immune consequences within sinonasal tissue beyond innate activation alone (43).

### 3.3 Depletion of protective tryptophan-derived metabolites

**Figure 2:**
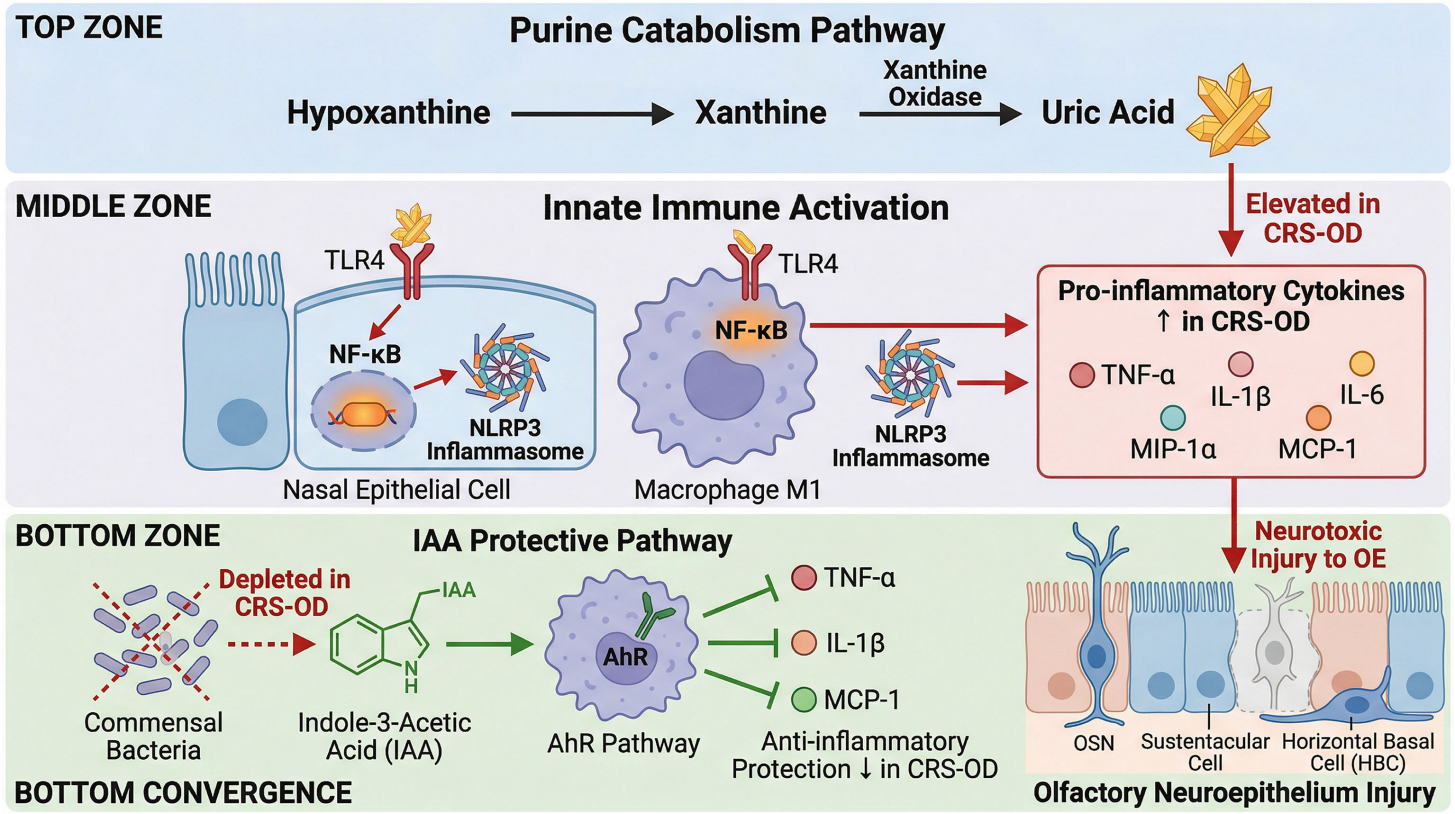
Dual metabolite-driven pathological axes mediating innate immune remodeling and olfactory neuroepithelial injury in the olfactory cleft niche. This schematic details the two core metabolite-driven pathological axes within the microbiota-metabolite-immune (MMI) axis that drive olfactory neuroepithelial (OE) damage in chronic rhinosinusitis with olfactory dysfunction (CRS-OD). The top zone delineates the dysregulated purine catabolism pathway in CRS-OD, where hypoxanthine is sequentially converted to xanthine and uric acid via xanthine oxidase, leading to pathological uric acid accumulation in the olfactory cleft (OC) mucus. The middle zone illustrates uric acid-mediated innate immune activation: uric acid acts as a damage-associated molecular pattern (DAMP) to engage Toll-like receptor 4 (TLR4) on nasal epithelial cells and resident macrophages, triggering downstream nuclear factor kappa-light-chain-enhancer of activated B cells (NF-κB) signaling and NOD-like receptor family pyrin domain containing 3 (NLRP3) inflammasome assembly. This cascade drives M1 macrophage polarization and robust production of pro-inflammatory cytokines and chemokines, including tumor necrosis factor-alpha (TNF-α), interleukin (IL)-6, IL-1β, macrophage inflammatory protein 1-alpha (MIP-1α), and monocyte chemoattractant protein-1 (MCP-1), which directly exert neurotoxic effects on the OE. The bottom zone depicts the impaired protective pathway mediated by indole-3-acetic acid (IAA), a tryptophan-derived metabolite produced by commensal bacteria. Indole-3-acetic acid (IAA) depletion in CRS-OD abrogates aryl hydrocarbon receptor (AhR)-dependent suppression of pro-inflammatory cytokine production in macrophages, eliminating local anti-inflammatory and neuroprotective defense and further exacerbating OE injury. The bottom convergence panel summarizes the final pathological outcome of these two axes: cumulative structural and functional damage to the OE, including injury to olfactory sensory neurons (OSNs), sustentacular cells, and horizontal basal cells (HBCs). Red arrows indicate disease-amplifying pathological processes; blue arrows indicate homeostatic protective processes disrupted in CRS-OD.

Beyond pathological metabolite accumulation, the CRS-OD metabolome is equally characterized by the loss of microbiome-derived protective molecules. Indole-3-acetic acid (IAA), a bacterial tryptophan catabolite produced by commensal upper respiratory and gut microbiota, was significantly depleted in OD patients compared to NOD controls (6). IAA exerts well-characterized anti-inflammatory effects by suppressing macrophage overproduction of TNF-alpha, IL-1beta, and MCP-1 through aryl hydrocarbon receptor (AhR)-dependent pathways (44–49). Its depletion within the olfactory niche may lower the activation threshold for innate immune responses to microbial products, contributing to a state of chronic neuroinflammation that may progressively compromise OSN function and survival. Applying OC mucus proteomics, *Guo* et al. and *Yoo* et al. additionally demonstrated downregulation of Lipocalin-1, Lipocalin-15, and odorant-metabolizing enzymes in chronic rhinosinusitis with nasal polyps (CRSwNP) patients, indicating that the molecular infrastructure for odorant processing within the OC mucus layer is broadly compromised under conditions of sustained dysbiosis (15, 33). Taken together, uric acid accumulation and IAA depletion provide a plausible two-component metabolic framework for understanding how dysbiosis might influence inflammation within the olfactory niche. The complete cascade of these two pathways, from microbial metabolite dysregulation to innate immune remodeling and final neurotoxic injury to the olfactory neuroepithelium, is detailed in **Figure 2**.

### 3.4 Emerging roles of short-chain fatty acids, bile acids, and polyamines

While purine and tryptophan metabolism are central to the MMI axis, other microbial-derived metabolites also play critical roles in olfactory cleft homeostasis. Short-chain fatty acids (SCFAs), such as butyrate, are renowned for their potent anti-inflammatory properties and their ability to strengthen epithelial barrier integrity. In the dysbiotic olfactory niche, the depletion of SCFA-producing bacteria compromises these protective mechanisms. Similarly, secondary bile acids exhibit significant immunomodulatory capacities, and their alteration in upper airway disease further disrupts local immune tolerance. Polyamines, including spermine and agmatine, are essential for cellular repair and epithelial maintenance. In addition to the uric acid and IAA axes, a second tier of metabolic dysregulation within the olfactory niche involves the depletion of short-chain fatty acids (SCFAs), secondary bile acids, and polyamines. Each of these metabolic perturbations independently amplifies mucosal inflammation and promotes olfactory epithelial injury, yet their effects ultimately converge on a shared pathological trajectory (50–54). Within the MMI axis framework, these metabolite perturbations constitute a second tier of the metabolic arm (Arm 2), operating in parallel with the uric acid and IAA axes. Their depletion or dysregulation reflects the collapse of commensal microbiota-dependent metabolic protection (Arm 1 dysbiosis) and directly amplifies the innate and adaptive immune dysregulation (Arm 3) through mechanisms including impaired epithelial barrier integrity, reduced AhR-mediated anti-inflammatory signaling, and disrupted cellular repair pathways within the olfactory neuroepithelium. Restoring these metabolite pools through targeted microbiome intervention therefore represents an underexplored but mechanistically coherent strategy within the MMI axis therapeutic framework.

## 4 *Staphylococcus aureus* superantigens and olfactory barrier disruption

### 4.1 Prevalence and pathological significance of S. aureus in CRS-OD

Within the MMI axis framework, *Staphylococcus aureus* is best viewed as an important pathobiont rather than the sole determinant of disease. Its overlapping superantigenic, biofilm-forming, and barrier-disrupting activities link and reinforce all three arms of the axis in CRS-OD. *S. aureus* simultaneously represents a consequence of olfactory niche dysbiosis and an active driver of the immune and metabolic dysregulation described in preceding sections. Its central pathobiological position in CRSwNP is reflected by nasal colonization rates reaching 67%, compared with approximately 27 to 33% in chronic rhinosinusitis without nasal polyps (CRSsNP) patients and healthy controls (55, 56). Specific Immunoglobulin E (IgE) antibodies against staphylococcal enterotoxins A and B (SEA/SEB) are detectable in approximately 50% of nasal polyp tissue homogenates from CRSwNP patients, and their presence independently correlates with disease severity, eosinophilic infiltration, and elevated systemic IgE (55, 57). A retrospective study by *Kim M* et al. in 388 subjects confirmed that SEA/SEB seropositivity was significantly more prevalent in CRSwNP than in CRSsNP or healthy controls, and that serum SE levels negatively correlated with objective olfactory function scores alongside blood eosinophil percentage and asthma comorbidity (58).

### 4.2 Superantigen-driven Th2 polarization and impairment of olfactory neurogenesis

Staphylococcal superantigens bypass conventional antigen presentation by cross-linking T cell receptor (TCR) Vβ regions with Major Histocompatibility Complex (MHC) class II molecules, triggering massive polyclonal T cell expansion and cytokine release (57, 59). In CRSwNP, superantigen exposure has been associated with a type 2-skewed cytokine milieu characterized by IL-4, IL-5, and IL-13, eosinophilic inflammation, and local IgE production. Furthermore, there is also evidence that S. aureus enterotoxins may alter regulatory T-cell function(Treg), although the extent of this effect in OC-specific tissue remains unclear. Superantigens can downregulate FOXP3 expression and reprogram Tregs into a pro-inflammatory phenotype, thereby breaking local immune tolerance and allowing unchecked inflammatory cascades to damage the olfactory epithelium (60). The Th2-skewed olfactory microenvironment directly impairs OSN renewal. In a rigorous murine model combining house dust mite extract and SEB sensitization over 22 weeks, *Rouyar* et al. demonstrated that type 2/Th2-mediated allergic CRS was associated with a significant reduction in the number and renewal rate of immature OSNs in the OE, even in the absence of measurable gross olfactory behavioral loss, suggesting that Th2 inflammation impairs the regenerative reserve of the OE before clinically apparent anosmia develops (8). These findings suggest that chronic Th2 inflammation may reduce the regenerative reserve of the olfactory epithelium before overt behavioral smell loss becomes apparent.

The IL-4/IL-4Rα axis has since been shown to directly modulate olfactory neuroimmune signaling. *Hara* et al. demonstrated that IL-4 activates olfactory sensory neurons via IL-4Rα signaling, inducing olfactory loss in murine models independently of polyp burden, with attenuation of effects in IL-4Rα knockout mice (61). Chronic IL-13 expression targeted to mature OSNs in a doxycycline-inducible mouse model produced a time-dependent, regionally specific loss of neurons from the OE, accompanied by morphological activation and proliferation of horizontal basal cells. However, their differentiation was directed away from OSN lineages, ultimately generating a regionally aneuronal epithelial phenotype (62). Under conditions of chronic type 2 inflammation, HBC differentiation is suppressed and redirected toward respiratory epithelial metaplasia through alterations in retinoic acid metabolism, representing a potentially irreversible structural endpoint of prolonged Th2-driven olfactory inflammation (2, 63).

A recent Nature Communications study demonstrated that IL-17a produced by aging CD4+ T helper cells impairs OE regeneration and promotes respiratory metaplasia through mechanisms involving NF-κB signaling pathway activation and suppression of Notch and Wingless-related Integration Site (Wnt)-dependent neurogenesis pathways, broadening the spectrum of inflammatory cytokines capable of disrupting olfactory niche homeostasis beyond the Th2 axis (64). While Th2 cells and macrophages are central to this axis, other critical components of mucosal immunity also actively participate in olfactory niche remodeling. Type 2 innate lymphoid cells (ILC2s) function as pivotal early effectors of the type 2 cascade. These innate lymphoid cells are increasingly recognized as key orchestrators of type 2 inflammation, and are well known to be highly elevated in human airway type 2 inflammatory diseases including allergic rhinitis, chronic rhinosinusitis with nasal polyps, and asthma. In the olfactory niche, epithelial cells responding to dysbiotic microbial signals release alarmin cytokines including IL-25, IL-33, and TSLP. These innate lymphoid cells depend on GATA-3 and generate IL-5, IL-9, and IL-13 in response to epithelial alarmins such as IL-25, IL-33, and TSLP, positioning them as early effectors of the type 2 cascade that precede and amplify adaptive Th2 engagement. Nasal polyp tissue in CRS is particularly enriched in ILC2s relative to control mucosa, and this enrichment is most pronounced in the eosinophilic subtype. Critically, this ILC2 activation precedes and amplifies adaptive Th2 engagement. Nasal polyps from patients with CRS exhibit elevated levels of cysteinyl leukotrienes (CysLTs), IL-33, TSLP, and IL-4, with converging evidence from multiple studies demonstrating that ILC2s are markedly enriched in nasal polyps, particularly in eosinophilic polyps, relative to control tissue. Together, these findings suggest that ILC2s may contribute to CRS pathogenesis and that eosinophilic polyposis is ILC2-driven (65, 66).

Dendritic cells (DCs) represent a second critical layer of innate immune surveillance. As bone marrow-derived antigen-presenting cells, DCs bridge innate microbial sensing and adaptive T-cell activation. The onset and development of chronic rhinosinusitis involve a serious imbalance in immune regulation, and recent studies have shown that an increase in DCs in CRS and their function of shaping the nasal mucosal immune response may play an important role in the pathogenesis of CRS. Nasal DCs continuously sample dysbiotic microbiome constituents and superantigens, with murine nasal DCs demonstrated to present antigen to CD4+ T cells and migrate in response to live bacterial pathogens. Patients with CRS had a significantly lower frequency of BDCA-1+ DCs than normal individuals. In many of the human biopsy samples studied for DC subsets, microbial studies demonstrated a persistence of coagulase-negative staphylococci. This, combined with the observed reduction in BDCA-1+ DCs in CRS, supports the hypothesis that a reduction in DCs, the key antigen presenting cells within the nose, may predispose to impaired pathogen clearance and chronic inflammation.

Additionally, in mixed or non-type 2 inflammatory endotypes, neutrophils are recruited by IL-17 and local chemokines and represent a numerically substantial cell population. Multiple studies have reported elevated proteolytic activity of elastase and cathepsin G, two granule-derived proteases released upon neutrophil activation, in the tissue of CRSwNP patients. Once secreted, elastase and cathepsin G are able to enhance secretion and activation of IL-1 family cytokines such as IL-1β, IL-33, and IL-36γ. Substrates for neutrophil proteases including elastin, collagen, and fibronectin are major components of the extracellular matrix, and neutrophil serine proteases exert a direct negative effect on the nasal epithelial barrier integrity. This proteolytic barrier disruption further amplifies antigen and pathobiont translocation into the olfactory subepithelium, directly linking neutrophilic activity to the dysbiosis-perpetuating arm of the MMI axis (65, 67, 68).

### 4.3 Biofilm-associated superantigen release and epithelial barrier disruption

**Figure 3:**
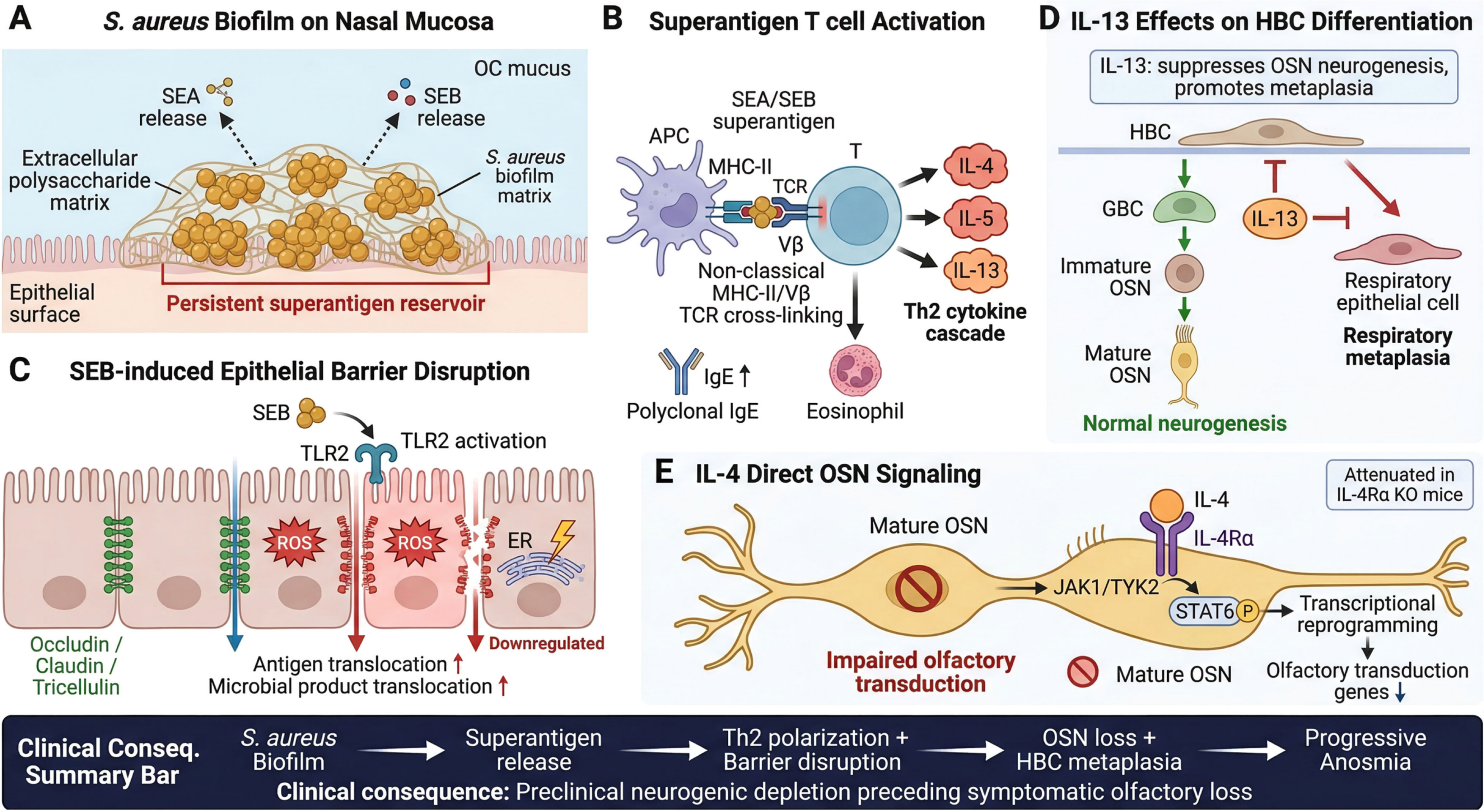
*Staphylococcus aureus* superantigen-mediated mechanisms of olfactory cleft barrier disruption and neurogenic impairment. This figure depicts the multifaceted pathogenic roles of *Staphylococcus aureus* (*S. aureus*) in linking and reinforcing all three arms of the MMI axis in CRS-OD. **(A)** Schematic of *S. aureus* biofilm formation on the sinonasal mucosal surface, with sustained release of superantigens (staphylococcal enterotoxin A/B, SEA/SEB) into OC mucus. **(B)** Superantigen-mediated non-canonical T cell activation via cross-linking of major histocompatibility complex (MHC) class II molecules and T cell receptor (TCR) Vβ regions, driving a T helper 2 (Th2) cytokine cascade (IL-4, IL-5, IL-13). **(C)** Staphylococcal enterotoxin B (SEB)-induced Toll-like receptor 2 (TLR2) activation, reactive oxygen species (ROS) generation, and downregulation of tight junction proteins (occludin, claudin) in nasal epithelial cells, leading to barrier disruption. **(D)** T helper 2 (Th2) cytokine-mediated impairment of olfactory neurogenesis, where IL-13 promotes respiratory metaplasia of horizontal basal cells (HBCs) and suppresses differentiation into OSNs. **(E)** Interleukin-4 (IL-4) mediated direct signaling on OSNs via the interleukin-4 receptor alpha (IL-4Rα) axis, impairing olfactory signal transduction. The bottom panel summarizes the clinical consequence of progressive OSN depletion and aneuronal epithelial transformation. Red arrows indicate pathological mechanisms; blue arrows indicate homeostatic processes disrupted by *S. aureus* superantigens. CRS-OD, chronic rhinosinusitis with olfactory dysfunction; MMI, microbiota-metabolite-immune; OC, olfactory cleft; OSN, olfactory sensory neuron.

Biofilms formed by *Staphylococcus aureus* within the sinonasal cavity may act as a reservoir for sustained superantigen exposure. The presence of *S. aureus* biofilms in patients with CRS is independently associated with eosinophilic inflammation, elevated eosinophil cationic protein (ECP) and IL-5, and Th2 skewing of the adaptive immune response, over and above the superantigenic effect of enterotoxin-specific IgE induction (69, 70). In parallel, experimental studies have shown that staphylococcal enterotoxin B (SEB) activates TLR2 signaling and triggers reactive oxygen species (ROS) generation and endoplasmic reticulum stress, ultimately disrupting epithelial cell integrity and enhanced paracellular permeability (55). The extracellular proteins and proteinases of *S. aureus* downregulate key tight junction proteins including occludin, claudin, and tricellulin in polarized nasal epithelial cells, facilitating greater antigen and microbial product translocation into the lamina propria and increasing exposure of OSN axonal projections to inflammatory stimuli (71–74). Staphylococcal superantigens additionally activate B cells to upregulate IL-4, IL-5, and IL-13 production, further perpetuating the type 2 inflammatory cycle within the olfactory niche (75). The multifaceted pathogenic roles of *S. aureus* in linking and reinforcing all three arms of the MMI axis, from biofilm formation and superantigen secretion to epithelial barrier disruption, Th2 inflammation, and impaired olfactory neurogenesis, are systematically depicted in **Figure 3**.

Collectively, these mechanisms form a self-amplifying vicious cycle that interconnects all three arms of the MMI axis. *Staphylococcus aureus* biofilm formation establishes a persistent reservoir of pathobiont colonization within the sinonasal compartment, displacing protective commensals and reducing microbial diversity. Continuous superantigen secretion from this biofilm reservoir drives Th2 polarization and polyclonal IgE production, generating a cytokine-rich local environment defined by elevated IL-4, IL-5, and IL-13. This Th2-dominated milieu further suppresses innate antimicrobial defenses, reduces the selective fitness of commensal genera such as *Corynebacterium* and *Dolosigranulum*, and degrades epithelial barrier integrity through tight junction protein downregulation. Increased mucosal permeability in turn amplifies pathobiont exposure and microbial product translocation into the subepithelial compartment, reinforcing dysbiosis and completing the vicious cycle. At the metabolite level, biofilm-associated purine catabolism contributes to uric acid accumulation within OC mucus, further engaging the TLR4/NF-κB innate immune activation arm of the MMI axis and converging on direct OSN cytotoxicity. This integrated perspective positions *S. aureus* not as a discrete pathological entity but as the principal biological node that simultaneously reinforces and links all three arms of the MMI axis in CRS-OD.

The clinical relevance of this cascade is supported by OC cytokine profiling. *Wu* et al. demonstrated that OC mucus Th2 cytokines IL-5 and IL-13 were significantly elevated in CRSwNP patients, and that mucus IL-5 levels correlated negatively with objective Threshold, Discrimination, and Identification (TDI) olfactory function scores across all CRS subjects (76). *Soler* et al. identified distinct OC biomarker-based inflammatory endotypes in CRS, with the highest olfactory burden occurring in clusters characterized by combined eosinophilic and putative *S. aureus*-associated inflammatory signatures (77).

### 4.4 Bidirectional interactions: immune alterations and epithelial injury driving dysbiosis

The relationship between the immune system and the microbiome in the olfactory cleft is highly bidirectional. Type 2 immune alterations, particularly elevated IL-4 and IL-13, directly suppress the production of endogenous antimicrobial peptides by nasal epithelial cells. This reduction in antimicrobial defense creates a permissive environment for pathobiont overgrowth. Concurrently, epithelial injury and the breakdown of tight junctions lead to the leakage of plasma proteins and nutrient-rich exudates into the mucosal lumen. This reciprocal relationship may help explain why dysbiosis and inflammation often coexist and persist in CRS, although the temporal sequence may differ across patients and endotypes (32).

## 5 Therapeutic strategies targeting the mmi axis for restoring olfactory homeostasis

### 5.1 Rationale and MMI axis-targeted paradigm

If microbial and metabolic perturbations contribute to olfactory dysfunction in CRS, then therapeutic strategies targeting the olfactory cleft microenvironment may complement conventional anti-inflammatory and surgical approaches. Interventions can target different nodes of the MMI axis: from the microbial end (e.g., topical sinonasal irrigations with xylitol or probiotics to restore eubiosis) to the immune end (e.g., biological agents like dupilumab). Topical sinonasal irrigations offer the most logistically feasible and anatomically targeted route for delivering microbiota-modifying agents directly to the olfactory niche, with the potential to reduce pathobiont colonization, restore commensal communities, modify local metabolite profiles, and thereby dampen the MMI axis-driven inflammatory cycle (78). We systematically summarized the key clinical studies investigating microbiota-targeted, metabolite-modulating, and immune-targeted therapies for CRS, with a focus on their documented or potential effects on olfactory function and sinonasal microbial homeostasis in **Table 3**.

**Table 3:** Clinical studies of microbiota-targeted and immune-targeted therapies for CRS: focus on olfactory function outcomes and microbiome regulation.

Study (author, year)	Journal	Intervention	Study design	n	Primary outcome	Effect on olfactory function	Effect on microbiome/S. aureus	Key limitations
Jiang RS et al. (2024) (83)	Biomedicines	Xylitol nasal irrigation post-FESS vs saline	RCT	70 (35 per arm)	Endoscopic score, olfactory threshold	Significant improvement in olfactory threshold	Significant reduction in S. aureus prevalence in nasal secretions	Short follow-up; no endotype stratification
Kang YJ et al. (2026) (84)	Clin Otolaryngol	Xylitol nasal irrigation vs saline	Systematic review and meta-analysis	263 (7 studies)	Sino-Nasal Outcome Test (SNOT), Nasal Obstruction Symptom Evaluation (NOSE), olfactory function, endoscopic score	Significant improvement in sinonasal well-being (SMD 0.6253, 95% CI 0.0067 to 1.2438)	Not assessed	Heterogeneous study designs; limited objective outcome data
Lambert PA et al. (2021) (78)	Laryngoscope Investig Otolaryngol	Xylitol or L. lactis irrigation post-FESS vs saline	Crossover	35 (25 CRS, 10 controls)	Microbiome diversity, SNOT-22	No significant change in SNOT-22	Increased Lactococcus detection after L. lactis use; no overall diversity change	Small sample; short 28-day treatment window
Lin L et al. (2017) (79)	Am J Otolaryngol	Xylitol nasal irrigation vs saline	RCT	76	SNOT-20, VAS	Significant improvement in subjective symptoms	Not assessed	No microbiome characterization
Mullol J et al. (2022) (87)	JACI Pract	Dupilumab 300 mg q2w (SINUS-24/52)	Phase 3 RCT	724	Loss of smell score, UPSIT	Rapid and sustained improvement from day 3; anosmia reversal	Not assessed	Post hoc analysis; no microbiome data
Lane AP et al. (2025) (89)	Curr Med Res Opin	Dupilumab (SINUS-52 post hoc)	Post hoc analysis	282 with baseline smell impairment	UPSIT impairment category	Significant improvement across all UPSIT categories including anosmia	Not assessed	Post hoc design; correlation with microbiome effects unknown
Anastasi F et al. (2025) (90)	Front Allergy	Dupilumab real-world	Retrospective observational	Multicenter Italian cohort	VAS olfactory, SS-I, NPS	Improvement in VAS and SS-I; NPS reduction did NOT correlate with SS-I recovery	Not assessed	Retrospective; no multi-omics data

This table summarizes clinical trials, systematic reviews, and observational studies investigating microbiota-targeted, metabolite-modulating, and immune-targeted therapies for CRS, with a focus on their documented effects on olfactory function and sinonasal microbial homeostasis. CRS, chronic rhinosinusitis; FESS, functional endoscopic sinus surgery; RCT, randomized controlled trial; SMD, standardized mean difference; CI, confidence interval; SNOT, Sino-Nasal Outcome Test; NOSE, Nasal Obstruction Symptom Evaluation; UPSIT, University of Pennsylvania Smell Identification Test; VAS, visual analogue scale; SS-I, Smell Screening Identification; NPS, nasal polyp score.

### 5.2 Xylitol nasal irrigation

Xylitol is a naturally occurring polyalcohol sugar alcohol with multifaceted antimicrobial, anti-biofilm, and immunomodulatory properties. Acting by osmosis on the nasal mucosal surface, xylitol reduces chloride concentration in the airway surface liquid (ASL), thereby restoring the activity of endogenous antimicrobial peptides including lysozyme, lactoferrin, and beta-defensins whose bactericidal efficiency is inversely proportional to local salt concentration (79, 80). Xylitol additionally exerts direct antibacterial effects by inhibiting bacterial glucose-dependent cell wall transport and glycolysis, and impairs biofilm formation by *S. aureus* and *Pseudomonas aeruginosa* at concentrations achievable by topical application (81, 82). A prospective randomized controlled trial by *Jiang* et al. demonstrated that xylitol nasal irrigation after functional endoscopic sinus surgery (FESS) significantly reduced endoscopic scores and improved olfactory threshold compared to pre-irrigation values, and significantly decreased the prevalence of *S. aureus* in nasal secretions (83). These findings are consistent with the possibility that xylitol can modify the microbiological composition of the olfactory niche in a manner predicted to reduce superantigen burden and interrupt the *S. aureus*-Th2-OD pathological cascade. A 2026 systematic review and meta-analysis by *Kang* et al. in Clinical Otolaryngology pooling seven studies with 263 participants confirmed that xylitol nasal irrigation significantly improved sinonasal well-being compared to saline irrigation (Standardized Mean Difference (SMD) 0.6253, 95% Confidence Interval (CI) 0.0067 to 1.2438, I² = 0.0%), though objective structural outcomes did not yet reach statistical significance, reflecting current evidence heterogeneity (84).

However, the available evidence supporting xylitol nasal irrigation has critical methodological limitations that warrant cautious interpretation. Published randomized controlled trials are characterized by small sample sizes, single-center designs, and follow-up durations typically ranging from four to twelve weeks. None of the included studies employed concurrent microbiome profiling to confirm that microbiological changes observed in nasal secretions translated into compositional shifts within the olfactory niche specifically. Furthermore, objective olfactory function was evaluated as a secondary rather than primary outcome in available trials, and no study stratified participants by CRS endotype. These limitations preclude definitive conclusions regarding the olfactory benefit of xylitol irrigation in the eosinophilic CRS subgroup most strongly associated with OD, and underscore the need for endotype-stratified trials with dedicated olfactory cleft sampling.

### 5.3 Probiotic irrigation and commensal restoration

Direct topical delivery of probiotic organisms to the sinonasal cavity represents a complementary strategy to restore commensal communities depleted in CRS-OD. *Lambert* et al. conducted a crossover microbiome study examining the effects of *Lactococcus lactis* W136 or xylitol irrigation in post-surgical patients with CRS using 16S rRNA gene sequencing, and demonstrated that L. lactis irrigation produced a detectable engraftment of *Lactococcus* within the sinonasal microbiome, providing proof of concept for probiotic delivery in this compartment (78). Future strain selection may reasonably prioritize organisms capable of competing with *S. aureus*, generating potentially protective metabolites, and supporting epithelial barrier integrity. Certain microbial genera known to catabolize uric acid and purine intermediates under anaerobic conditions represent additional rational candidates for lowering OC uric acid burden via the microbiome (35, 45, 85).

Despite this proof-of-concept, the clinical translation of topical probiotic therapy to the olfactory niche faces considerable technical and safety challenges. Sustained engraftment of exogenous probiotic strains within the sinonasal microbiome beyond the active treatment period has not been demonstrated, and the optimal delivery vehicle, dosing frequency, and strain selection for olfactory niche-specific restoration remain undefined. The proximity of the olfactory niche to the cribriform plate and the olfactory bulb raises theoretical concerns regarding the potential for ascending infection in immunocompromised patients, and the absence of long-term safety data in post-surgical sinonasal cavities requires prospective evaluation before routine clinical application can be considered (86).

### 5.4 Biological agents: targeting Th2 immunity with implications for the MMI axis

Dupilumab, a fully human monoclonal antibody targeting the shared IL-4/IL-13 receptor subunit IL-4Rα, has demonstrated the most robust and consistent improvement in olfactory function among currently approved biological therapies for CRSwNP. In the phase 3 SINUS-52 trial, dupilumab produced rapid and progressive improvement in loss of smell scores evident by day 3 of treatment, with effects sustained through 52 weeks (87, 88). *Post hoc* analysis confirmed dupilumab significantly improved University of Pennsylvania Smell Identification Test (UPSIT) scores across all impairment categories including baseline anosmia, suggesting that mechanisms of olfactory recovery extend beyond simple polyp reduction (89). A real-world observational study found no correlation between nasal polyp score reduction and objective smell recovery under dupilumab, indicating that cytokine-level effects at the OSN and progenitor cell level account for a substantial component of the olfactory benefit (90).

Mechanistically, the clinical effect of dupilumab is consistent with experimental evidence linking IL-4/IL-13 signaling to olfactory neuronal dysfunction, impaired neurogenesis and barrier abnormalities. By contrast, the idea that dupilumab secondarily reshapes the olfactory microbiome remains plausible but unproven (71, 91, 92).

### 5.5 Impact of antibiotic use and antimicrobial resistance

The widespread use of systemic and topical antibiotics in CRS management introduces significant secondary disruptions to the MMI axis. Repeated antibiotic exposure severely depletes the protective commensal microbiota, leading to a long-term reduction in microbial diversity within the olfactory cleft. This ecological void facilitates the colonization and expansion of multidrug-resistant pathobionts, such as methicillin-resistant *Staphylococcus aureus* (MRSA) and resistant *Pseudomonas aeruginosa* strains. These ecological consequences may be relevant to the olfactory cleft, although direct evidence remains limited. The persistence of these resistant biofilms not only leads to treatment failure but also perpetuates the chronic inflammatory signaling that drives olfactory dysfunction. Consequently, antimicrobial stewardship and microbiome-sparing therapies are critical for preserving olfactory niche homeostasis (32, 93, 94).

**Figure 4:**
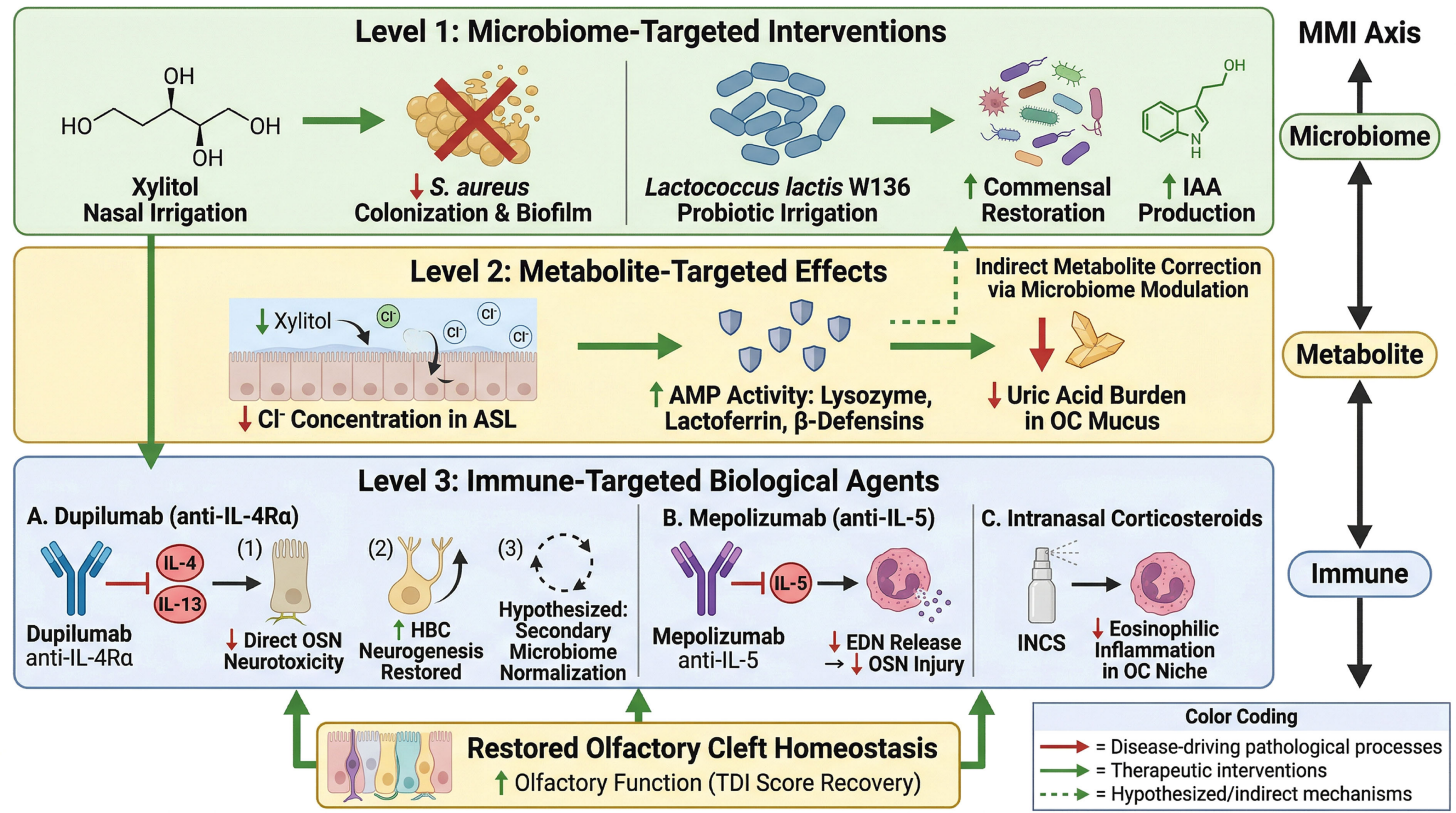
Hierarchical therapeutic framework targeting the microbiota-metabolite-immune axis to restore olfactory homeostasis in CRS-associated olfactory dysfunction. This schematic presents a tiered therapeutic framework targeting the three core arms of the microbiota-metabolite-immune (MMI) axis to reverse pathological cascades and restore olfactory homeostasis in chronic rhinosinusitis with olfactory dysfunction (CRS-OD). Level 1 (Microbiome-Targeted Interventions) focuses on correcting microbial dysbiosis, the initiating arm of the MMI axis: xylitol nasal irrigation inhibits *Staphylococcus aureus* colonization and biofilm formation, while topical probiotic irrigation with *Lactococcus lactis* W136 restores depleted commensal microbial communities and rescues the production of protective metabolites such as indole-3-acetic acid (IAA). Level 2 (Metabolite-Targeted Modulation) addresses the intermediate metabolic arm of the MMI axis: xylitol reduces chloride concentration in the airway surface liquid (ASL), enhancing the bactericidal activity of endogenous antimicrobial peptides (AMPs) and indirectly lowering the burden of neurotoxic purine metabolites (notably uric acid) in the olfactory cleft (OC) microenvironment. Level 3 (Immune-Targeted Therapeutics) targets the downstream immune effector arm of the MMI axis: dupilumab, a fully human anti-interleukin-4 receptor alpha (IL-4Rα) monoclonal antibody, blocks IL-4/IL-13-mediated direct neurotoxic signaling on olfactory sensory neurons (OSNs), reverses IL-13-induced suppression of horizontal basal cell (HBC) neurogenic differentiation, and dampens type 2 inflammatory cascades, with a hypothesized secondary effect of normalizing the OC microbiome by interrupting the bidirectional dysbiosis-inflammation vicious cycle. Additional immune-targeted strategies include mepolizumab (anti-IL-5 monoclonal antibody), which reduces eosinophil-derived neurotoxin (EDN) release, and intranasal corticosteroids, which attenuate local eosinophilic inflammation in the OC niche. Red pathways indicate disease-driving processes in the MMI axis; green pathways indicate therapeutic interventions and their downstream homeostatic effects.

The olfactory benefits of dupilumab described above represent robustly demonstrated clinical outcomes derived from phase 3 randomized trial data. In contrast, the hypothesis that dupilumab may secondarily reshape the olfactory niche microbiome constitutes a biologically plausible but currently unverified extension of these findings, and this distinction warrants explicit acknowledgment. The mechanistic basis for such potential effects lies in the bidirectional relationship between type 2 inflammation and microbial dysbiosis within the olfactory niche. Th2 cytokines, particularly IL-4 and IL-13, suppress innate epithelial antimicrobial defenses, reduce the selective fitness of commensal species, and generate a mucosal environment permissive to pathobiont outgrowth including *S. aureus (*75). Conversely, pathobiont-driven superantigen release amplifies Th2 polarization, further reinforcing the dysbiotic state (55, 57). By blocking the shared IL-4Rα subunit, dupilumab may interrupt this bidirectional vicious cycle: suppression of the Th2 cytokine environment could theoretically restore the selective pressure favoring commensal recolonization over pathobiont persistence, thereby indirectly normalizing microbiome composition and attenuating the microbial drivers of metabolite perturbation within the MMI axis. Recent data demonstrating dupilumab’s capacity to reduce local type 2 pro-inflammatory biomarkers in CRSwNP nasal polyp tissue supports the biological plausibility of such downstream effects (82). Whether this microbiome-reshaping effect operates in clinical practice remains the most critically important open question within the MMI axis field and warrants dedicated longitudinal multi-omics investigation in dupilumab-treated CRS cohorts.These emerging therapeutic strategies target different core arms of the MMI axis, from reversing microbial dysbiosis and correcting metabolite dysregulation to suppressing pathological immune activation, with the shared goal of restoring olfactory cleft homeostasis. The hierarchical therapeutic framework targeting the MMI axis, and their mechanisms of action for reversing the pathological cascade and restoring olfactory function, are summarized in **Figure 4**.

## 6 Future perspectives and research gaps

The MMI axis framework highlights several priorities for future research. Among these, the development of standardized olfactory cleft-specific sampling protocols stands as perhaps the most foundational requirement, since the validity of any downstream microbiome or metabolomic inference depends critically on sampling precision. The endoscopic Leukosorb strip technique, established for OC cytokine collection, should be systematically adapted for metagenomic and metabolomic applications in multicenter cohorts (95, 96).

Establishing causal mechanisms, rather than extending the current catalogue of associations, represents an equally urgent imperative. For instance, utilizing conditional knockout models, such as olfactory epithelium-specific TLR4 knockout mice (*TLR4−/−*), could definitively determine whether uric acid or Gram-negative endotoxin accumulation is the direct cause of OSN injury. Additionally, pharmacological interventions using specific URAT1 inhibitors (e.g., probenecid) in murine models of CRS could verify whether blocking uric acid uptake reverses local microenvironmental inflammation and rescues olfactory function. These approaches, combined with germ-free animal models colonized with defined olfactory niche microbiota, OSN electrophysiology, and single-cell transcriptomic profiling, are essential. Olfactory organoid platforms capable of modeling HBC-to-OSN differentiation trajectories under defined inflammatory and microbial metabolite conditions represent particularly tractable tools for mechanistic dissection (62, 64).

On the therapeutic horizon, the identification of purified uric acid-catabolizing microbiota as candidate therapeutics for the sinonasal niche deserves prioritized preclinical investigation. The demonstrated capacity of gut microbiota genera including *Lactobacillus* and *Akkermansia* to perform anaerobic purine catabolism with concurrent short-chain fatty acid production provides a mechanistic template for designing synbiotic preparations targeting the purine metabolic arm of the MMI axis (35, 45).

Finally, longitudinal multi-omics profiling of patients with CRS during dupilumab therapy will be essential to determine whether IL-4Rα blockade reshapes the olfactory niche microbiome composition alongside its documented effects on tissue cytokines, and whether microbiome normalization correlates with the degree of olfactory recovery. Fifth, the nasal-brain axis implications of olfactory niche dysbiosis demand integration of olfactory microbiome data with longitudinal neurocognitive outcomes in dedicated cohort studies. This is particularly relevant regarding early neuroinflammatory events predisposing to neurodegeneration. Restoring olfactory function through MMI axis-targeted therapies holds profound implications for overall health and quality of life (97). Clinical studies demonstrate that the recovery of smell significantly improves nutritional status by restoring flavor perception and appetite. Furthermore, olfactory improvement is strongly correlated with reduced rates of depression and enhanced cognitive well-being in patients with CRS, highlighting the systemic benefits of resolving olfactory cleft inflammation (98–101). Looking forward, the MMI axis framework may extend beyond CRS to other conditions characterized by olfactory loss. Olfactory dysfunction is a well-documented early prodromal marker for neurodegenerative diseases, including Alzheimer’s disease and Parkinson’s disease. Investigating whether dysbiosis and metabolite perturbations in the olfactory cleft serve as early triggers for central neuroinflammation could open new diagnostic and therapeutic avenues for these severe neurological disorders (27–29, 31).

A notable evidence gap concerns the role of the MMI axis in non-type 2 CRS endotypes, which are prevalent in Asian and pediatric populations and characterized by neutrophilic or mixed inflammatory infiltration with predominance of IFN-γ and IL-17 rather than Th2 cytokines (16, 25, 102, 103). In these endotypes, distinct microbiome compositions enriched in gram-negative organisms such as *Pseudomonas aeruginosa* and *Haemophilus influenzae* may engage the purine metabolite-TLR4 innate activation arm of the MMI axis through pathways that are mechanistically separable from superantigen-driven Th2 polarization. Available evidence from *Xu* et al. suggests that purine catabolism dysregulation modulates the Th17/Treg balance in non-eosinophilic nasal epithelial inflammation (40), raising the possibility that a modified MMI axis with overlapping metabolite-driven but immunologically distinct pathways operates in non-type 2 CRS-OD. Systematic multi-omics characterization of the olfactory niche in non-type 2 endotypes represents a high-priority research direction, as current evidence for the MMI axis is largely derived from eosinophilic or mixed CRS cohorts.

## 7 Conclusion

The evidence synthesized in this review makes clear that CRS-associated olfactory dysfunction cannot be adequately explained by mechanical obstruction or generic type 2 inflammation alone. Evidence summarized in the MMI axis framework presented in this review suggests that OD may be viewed as a disorder of the olfactory cleft microenvironment, in which pathobiont enrichment, metabolite dysregulation, particularly purine catabolism and IAA depletion and sustained immune activation converge to injure olfactory neuroepithelium and impair its regenerative capacity. Key mechanistic drivers include *Acinetobacter*-associated dysbiosis with commensal depletion, uric acid-mediated TLR4/NF-κB innate immune activation, and *S. aureus* superantigen-driven Th2 polarization with direct neurotoxic consequences for OSN progenitor cell biology. These factors are interconnected through a self-amplifying MMI vicious cycle in which each arm mutually reinforces the others. Emerging therapeutic strategies targeting this axis, ranging from topical xylitol and probiotic irrigation to IL-4Rα blockade with dupilumab, show mechanistic coherence and a growing clinical evidence base for olfactory benefit, while the current predominance of association-level data and the relative paucity of olfactory cleft-specific causal evidence define the foremost research priorities ahead. Precision multi-omics investigation of the olfactory niche, informed by the MMI axis framework, holds promise for transforming the treatment of CRS-OD from symptom management toward targeted restoration of olfactory homeostasis.
